# Motor and cognitive functioning in children treated for idiopathic clubfoot at the age of 3 years

**DOI:** 10.1186/s12887-019-1765-3

**Published:** 2019-10-30

**Authors:** Julia Dillmann, Gudrun Schwarzer, Christian-Dominik Peterlein

**Affiliations:** 1 Department of Developmental Psychology, Justus-Liebig-University, Otto-Behaghel-Str. 10F, 35394 Gießen, Germany; 2 Center for Orthopaedics and Trauma Surgery, University Hospital Giessen and Marburg, Baldingerstraße, 35043 Marburg, Germany

**Keywords:** Children, Congenital idiopathic clubfoot, Ponseti method, Cognitive development, Motor development, Bayley-III

## Abstract

**Background:**

Several studies have investigated motor and cognitive skills in infants as well as gross motor abilities in schoolchildren treated for congenital idiopathic clubfoot, mostly indicating specific impairments in those children. However, until now, little is known about the motor and cognitive abilities of preschool children treated for idiopathic clubfoot. Thus, it was the aim of this study to examine gross motor, fine motor and cognitive skills of 3-year-old-children treated for idiopathic clubfoot.

**Method:**

We tested gross motor, fine motor and cognitive functioning of 10 children treated for idiopathic clubfoot and 10 typically developing children at the age of 40 months (SD = 1) with the Bayley Scales of Infant and Toddler Development.

**Results:**

The children treated for idiopathic clubfoot showed a slight delay in gross motor development. In particular, they demonstrated difficulties in tiptoeing, walking upstairs and walking downstairs. Moreover, we found some slight deficits in cognitive development, particularly in visual-spatial memory.

**Discussion:**

Children treated for idiopathic clubfoot appear to have an increased risk of gross motor and spatial cognitive deficits. Orthopedic pediatrics should incorporate measures of gross motor functioning, for example tiptoeing, in their orthopedic setting. Moreover, future studies are needed to clarify whether the observed deficits persist through childhood. If so, some kind of a motor training for children with idiopathic clubfoot might be required.

## Background

Congenital idiopathic clubfoot, an isolated birth defect with multifactorial etiology [1–4] has a prevalence in Central Europe and North America of 1–2 in 1000 newborns [5, 6]. For approximately 50 %, idiopathic clubfoot is bilateral and males are affected more frequently than females [6, 7]. Typically, the foot is rotated and characterized by midfoot cavus, forefoot adductus and hindfoot equinovarus. Mostly, idiopathic clubfoot is corrected using the Ponseti method [8, 9], a predominantly conservative treatment with little operative interventions, good functional results and a relapse rate that is comparable to that of other treatments [10–13]. With the goal of sufficient foot mobility, treatment should start as soon as possible after birth. The Ponseti method consists of weekly manipulation and casting, percutaneous Achilles tenotomy (90% of patients) and foot abduction bracing [8, 9]. Previous studies in infants treated for idiopathic clubfoot revealed some gross motor impairments, especially in the second part of the first year of life, particularly in crawling and walking [14–17]. Additionally, there seems to be some slight deficits in specific cognitive tasks, including problem solving and spatial cognition in clubfoot infants [14], that parallels the corresponding link between motor and cognitive development in healthy infants [18–21] and infants with locomotor delay due to spina bifida [22]. Moreover, studies in older children treated for idiopathic clubfoot demonstrated an increased risk of motor activity limitations [23], gross motor deficits and asymmetrics [24] as well as a slightly decreased walking capacity [25]. In contrast, another study did not find any impaired athletic abilities in school-age children after satisfactory treatment of congenital clubfoot [26]. However, no studies have been made on motor and cognitive abilities in children treated for idiopathic clubfoot at the age of 3 years. Previous studies primary focused on motor development of infants or school-age children with mixed results regarding the long-term recovery from the Ponseti-treatment during infancy. Hence, the aims of this study were to investigate both motor and cognitive abilities among 3-year-old children treated for idiopathic clubfoot with the Bayley Scales of Infant and Toddler Development, third edition [27].

Based on previous studies [14–17], we expected that children treated for idiopathic clubfoot would be delayed in gross motor development but we did not expect to find any impact on fine motor development. According to a previous study [23], we did not expect any gross motor differences between children with unilateral and children with bilateral clubfoot. Following previous studies with children treated for spina bifida [22, 28], we expected that children treated for idiopathic clubfoot could show some deficits in spatial cognitive functioning.

## Method

### Ethics statement

The present study was conducted in full accordance with the Research Ethics Guidelines of the German Psychological Society (DGPs). The Office of Research Ethics at the University of Giessen approved the experimental procedure and the informed consent protocol. Moreover, all parents provided written informed consent prior to the first testing of their children.

### Participants

A total of 20 children participated in our study. All tested children were born at term and from middle class families. Ten children (1 female, 9 males), treated for congenital idiopathic clubfoot (6 unilateral, 4 bilateral), were matched concerning age and gender with 10 typically developing children. We had to exclude two additional clubfoot children from the study because they were born prior to the 37th week of pregnancy. Additionally, we had to exclude two clubfoot children because of missed appointments. The mean age of the clubfoot group was 40 months (range: 39–41, SD = 1). Those children had no further impairments and were recruited from the Department of Orthopaedics at the university hospital of Giessen and Marburg, Germany, where the clubfeet were treated according to the Ponseti method. The treatment of all participants with idiopathic clubfoot was the same, including weekly manipulation and casting (mean number of casts M = 5.44, SD = 1.59), an Achilles tendon tenotomy and foot abduction bracing. All patients had to wear the foot abduction orthosis full time during their first months of life, gradually reduced to only nighttime. At the age of 3 years, none of the patients had any relapse and all of them used braces at night. We randomly chose for every child treated for idiopathic clubfoot a typically developing child, matching gender and age, from the address lists of the department of Developmental Psychology at the University of Giessen, Germany. The typically developing children came to be on the address list of our department because they have already participated in an earlier study and agreed to be contacted again. Originally, we got the addresses from the city administration. The mean age of the control group was 40 months (range: 39–42, SD = 1).

### Measures

We measured gross motor, fine motor and cognitive skills of all children with a standardized and objective measuring instrument, the German version of the Bayley Scales of Infant and Toddler Development, third edition [27]. The Bayley-III-Scales are reliable, valid and applied measurement tools for assessing early development both in clinical practice and research settings (Bayley, 2006). The Bayley-III-Scales are based on direct child interaction, and the experimenter assess the performance of the child immediately during the testing. The items of the cognitive subscale measure sensorimotor development, exploration and manipulation, object relatedness, concept formation, memory and other aspects of cognitive processing. The items of the fine motor subscale quantify prehension, perceptual-motor integration, motor planning and motor speed. The items of the gross motor subscale assess static positioning; dynamic movement, including locomotion and coordination; balance; and motor planning [27]. We derived scaled scores from the subtests’ total raw scores. The Bayley-III scaled scores represent a child’s performance relative to same-age peers and range from 1 to 19, with a mean (*M*) of 10 and a standard deviation *(SD)* of 3 [27].

### Procedure

Written informed consent was obtained from all parents in advance. The two experimenters were non-blinded with regard to the clubfoot diagnosis of the children. However, they were carefully trained and supervised by experienced researchers regarding the administration and interpretation of the Bayley-III-Scales. This should ensure that they were able to run and to analyse the Bayley-III-Scales objectively, regardless of the children’s diagnosis. One assessor tested the children twice within 1 week, individually at home or in a laboratory at the university. Each testing session took approximately 1 h and was videotaped by another experimenter, giving the possibility for reanalyzing off-line. We measured gross motor and cognitive skills at the first testing session and fine motor skills at the second testing session. According to the manual [27], each subscale test started with an appropriate start item that corresponded with the chronological age of the child and continued until the child failed five consecutive items. If a child failed one of the first three items, the items corresponding with the previous age group were presented. Each participating child received a small toy as gift after the first and second test session as well as a certificate of participation after the second testing session.

### Data analysis

Initially, we converted the raw scores into scaled scores using the tables in the Bayley manual [27]. The normality of our data sets was assessed by the Kolmogorov–Smirnoff test of normality. Fine motor and cognitive data were normal distributed (*ps* > .05); however, not the gross motor data of the control group (*p* < .05). Hence, we conducted independent samples *t*-tests to analyze the fine motor and cognitive performances and a Mann–Whitney-U-test to analyze the gross motor performances of both groups. We further compared the performance of both groups on each item of the Bayley Scales individually with Fisher’s exact tests to uncover whether group differences exist on specific tasks. We chose Fisher’s exact test because of our small sample size. We used SPSS 22.0 [29] for all statistical analyses and G*Power 3.1 [30] for calculating effect sizes (Cohens *d*) and statistical power (1-*ß).*

## Results

### Gross motor development

**Fig. 1:**
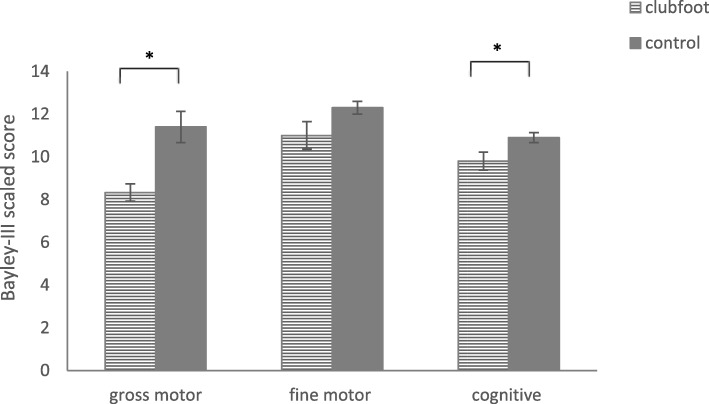
Mean Bayley scaled scores of all children treated for idiopathic clubfoot at 40 months of age. Error bars indicate the standard error of the mean, and an asterisk indicates statistical significance, *p* < .01

As can be seen from Fig. 1, the 3-years-old children treated for idiopathic clubfoot (*M* = 8.33, *SD* = 1.23) showed significantly lower gross motor performance than the typically-developing children (*M* = 11.40, *SD* = 2.32) at the same age (Mann–Whitney-U = 6.00, *p* = .001, *d* = 1.655, 1-*ß* = 0.965). Moreover, we did not find any significant differences between children with unilateral and children with bilateral clubfoot (*t* (7) = .171, *p* = .869).

**Fig. 2:**
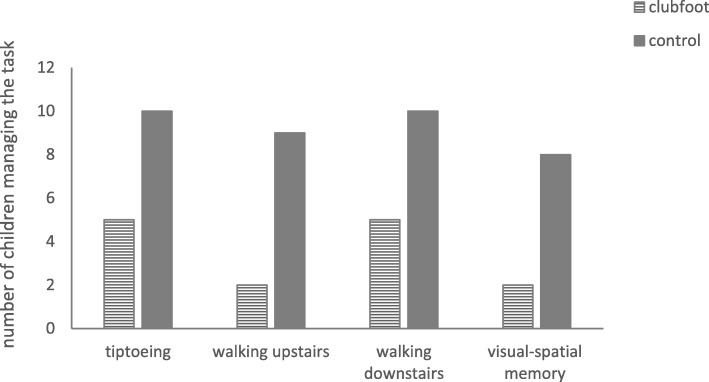
Significant differences (all *ps* < .05) between the two groups regarding individual gross motor and cognitive items

Notably, as can be seen from Fig. 2, children treated for idiopathic clubfoot had difficulties with tiptoeing (*p* = .033, Fisher’s exact test), walking upstairs (*p* = .005, Fisher’s exact test, *d =* 1.883), and walking downstairs (*p* = .033, Fisher’s exact test).

### Fine motor development

As seen from Fig. 1, the total fine motor performance of the children treated for idiopathic clubfoot (*M* = 11.00, *SD* = 1.85) was comparable (*t* (17) = − 1.935; *p* = .071, d = 0.884; 1-*ß* = 0.555) to those of the typically developing children (*M* = 12.30, *SD* = 0.95). Moreover, we did not find any significant differences concerning the performance on individual fine motor items (all *ps* > .05, Fisher’s exact test).

### Cognitive development

As seen in Fig. 1, clubfoot children (*M* = 9.80, *SD* = 1.32) showed significantly lower total cognitive performance (*t* (18) = − 2.305; *p* = .033, *d* = 1.04) than the typically-developing children (*M* = 10.90, *SD* = 0.74), especially in visual-spatial memory (*p* = .023, Fisher’s exact test). As can be seen from Fig. 2, children treated for idiopathic clubfoot were less able to select object pairs correctly, a task quantifying visual-spatial-memory performance. However, on all other cognitive items, the two groups achieved comparable results.

## Discussion

This is, to our knowledge, the first study investigating motor and cognitive abilities of 40-months old children treated for idiopathic clubfoot. The current study yielded several important results and implications:

First, 3-years old children treated for idiopathic clubfoot appear to have an increased risk of gross motor limitations. In particular, we found deficits in tiptoeing, walking upstairs and walking downstairs without support. Indeed, the children treated for idiopathic clubfoot were able to walk upstairs/downstairs, however, they did not manage to perform the special requirement for these tasks, for example needing to hold on to the handrail. Hence, orthopedic pediatrics should incorporate measures of gross motor functioning, for example tiptoeing, in their orthopedic setting to uncover children with a need for further diagnostic and therapy. Moreover, consistent with previous findings [23], children with unilateral clubfoot showed the same gross motor results as children with bilateral clubfoot.

Second, as expected, the present results demonstrated age-appropriate fine motor performances in 40-months old children with idiopathic clubfoot. Our findings confirmed previous findings in infants treated for idiopathic clubfoot [14, 31].

Third, we found some slight cognitive impairments in children treated for idiopathic clubfoot. These children had difficulties selecting object pairs correctly, a task quantifying visual-spatial-memory performance. Particularly, the children were allowed to view 10 s a display of six cards (three object pairs: tops, flowers and cars). Then, the cards were turned over and the children had to identify the correct pairs of cards for the first two objects (tops and flowers) [27]. Notably, the children treated for idiopathic clubfoot had difficulties selecting these object-pairs correctly. However, on all other cognitive items, the two groups achieved comparable results. Hence, it seems that gross motor development does not have a strong impact on cognitive development per se, but it seems to facilitate the development of specific cognitive skills, for example visual spatial memory performance. These results confirmed the findings of current studies in typically developing children [18–21], spina bifida children [22] and infants treated for idiopathic clubfoot [14]. Overall, it seems that gross motor abilities can influence specific spatial cognitive skills, emphasizing the need for a motor intervention program for children with strong motor disabilities.

The major strengths of this study were definitely the testing of both, motor and cognitive abilities in children treated for idiopathic clubfoot as well as in typically developing children. One former study did not test a group of healthy control children, instead they simply compared the values of children treated for idiopathic clubfoot with norm-based values [23]. The major limitation of the present study is definitely the small sample size, only 20 children, 10 typically developing and 10 treated for idiopathic clubfoot, participated in this study. However, despite the relatively small sample size, the analyses revealed significant differences with large effect sizes and high statistical power [32], indicating considerable differences between the two groups. Other obvious limitations of the current study are the non-blinding of the experimenter and the matching procedure: We matched the children of the two groups only concerning age and gender. We were not able to match other attributes such as weight or height. Another limitation was that the two experimenters were not blinded with regard to the diagnosis of the children. However, the Bayley III-Scales are objective, reliable, valid and applied measurement tools for assessing early motor and cognitive development both in clinical practice and research settings [27]. Therefore, we consider it unlikely that the data were considerably influenced by subjective factors of the experimenters.

Initially, future studies should investigate motor and cognitive abilities of children treated for idiopathic clubfoot with a larger sample to verify our results. Moreover, previous studies have shown that school-aged children with motor difficulties due to spina bifida or overweight demonstrated impaired spatial cognitive skills [28, 33]. Hence, future studies in older children treated for idiopathic clubfoot are needed to examine whether the observed gross motor and spatial cognitive deficits persist through childhood. If so, some kind of motor intervention program might be required. Finally, previous studies revealed that motor competence seems to be important for school children’s self-esteem [34, 35]. Hence, future studies with school-aged children treated for idiopathic clubfoot should measure self-esteem additionally.

## Conclusions

This study showed that 40-months old children treated for idiopathic clubfoot appear to have an increased risk of specific gross motor and spatial cognitive impairments. Our results indicate that gross motor difficulties can affect spatial cognitive skills in children treated for idiopathic clubfoot. Therefore, orthopedic pediatrics should incorporate measures of gross motor functioning, for example tiptoeing, in their orthopedic setting to uncover children with a need for further diagnostic and therapy.

## Data Availability

The datasets used and analyzed during the study are available on reasonable request to the corresponding author.
